# Electroanalytical Performance of Nitrogen-Doped Graphene Films Processed in One Step by Pulsed Laser Deposition Directly Coupled with Thermal Annealing

**DOI:** 10.3390/ma12040666

**Published:** 2019-02-23

**Authors:** Florent Bourquard, Yannick Bleu, Anne-Sophie Loir, Borja Caja-Munoz, José Avila, Maria-Carmen Asensio, Gaëtan Raimondi, Maryam Shokouhi, Ilhem Rassas, Carole Farre, Carole Chaix, Vincent Barnier, Nicole Jaffrezic-Renault, Florence Garrelie, Christophe Donnet

**Affiliations:** 1 Laboratoire Hubert Curien, UMR 5516 CNRS, Université de Lyon, Université Jean Monnet, F-42000 Saint-Étienne, France; Florent.Bourquard@univ-st-etienne.fr (F.B.); yannick.bleu@univ-st-etienne.fr (Y.B.); Anne.Sophie.Loir@univ-st-etienne.fr (A.-S.L.); Florence.Garrelie@univ-st-etienne.fr (F.G.); 2 Synchrotron SOLEIL, Université Paris-Saclay, Saint Aubin, F-91192 Gif sur Yvette, France; borja.caja-munoz@synchrotron-soleil.fr (B.C.-M.); jose.avila@synchrotron-soleil.fr (J.A.); 3 Instituto de Ciencia de Materiales de Madrid, Sor Juana Inés de la Cruz, 328049 Madrid, Spain; mc.asensio@csic.es; 4 Institut des Sciences Analytiques, UMR 5280 CNRS, Université de Lyon, Université Claude Bernard Lyon 1, F-69100 Villeurbanne, France; gaetan.raimondi@isa-lyon.fr (G.R.); m.shokouhi89@gmail.com (M.S.); ilhemras@hotmail.fr (I.R.); carole.farre@univ-lyon1.fr (C.F.); carole.chaix-bauvais@univ-lyon1.fr (C.C.); Nicole.Jaffrezic@univ-lyon1.fr (N.J.-R.); 5 Mines Saint-Etienne, Université de Lyon, UMR 5307 CNRS, Centre SMS, F-42023 Saint-Etienne, France; barnier@emse.fr

**Keywords:** graphene, nitrogen-doped graphene, pulse laser deposition, electrochemical analysis, oxygen peroxide oxidation

## Abstract

Graphene-based materials are widely studied to enable significant improvements in electroanalytical devices requiring new generations of robust, sensitive and low-cost electrodes. In this paper, we present a direct one-step route to synthetize a functional nitrogen-doped graphene film onto a Ni-covered silicon electrode substrate heated at high temperature, by pulsed laser deposition of carbon in the presence of a surrounding nitrogen atmosphere, with no post-deposition transfer of the film. With the ferrocene methanol system, the functionalized electrode exhibits excellent reversibility, close to the theoretical value of 59 mV, and very high sensitivity to hydrogen peroxide oxidation. Our electroanalytical results were correlated with the composition and nanoarchitecture of the N-doped graphene film containing 1.75 at % of nitrogen and identified as a few-layer defected and textured graphene film containing a balanced mixture of graphitic-N and pyrrolic-N chemical functions. The absence of nitrogen dopant in the graphene film considerably degraded some electroanalytical performances. Heat treatment extended beyond the high temperature graphene synthesis did not significantly improve any of the performances. This work contributes to a better understanding of the electrochemical mechanisms of doped graphene-based electrodes obtained by a direct and controlled synthesis process.

## 1. Introduction

Graphene is considered to be a promising 2D material thanks to its unique versatile properties, in particular high thermal and electrical conductivity, with many potential applications as an electrode in chemistry and as an electrocatalyst in fuel cells, field emitters, batteries, supercapacitors, and sensors (for a review, see Reference [1]).

The structure and electronic properties of graphene can be tailored by heteroatom doping, in particular by incorporating nitrogen, thereby opening the band gap and transforming graphene into a semiconductor, as recently reviewed by Yadav et al. [2] and Xu et al. [3].

Some authors have studied the electrochemical properties of N-doped graphene (NG) in various experimental conditions. Wang et al. [4] obtained NG with a nitrogen content ranging between 0.11 and 1.35 at %, by nitrogen plasma exposure of graphene prepared by chemical reduction of graphene oxide. The NG films exhibited consistent electrocatalytic activity for the reduction of hydrogen peroxide (H_2_O_2_), as well as high sensitivity and selectivity for glucose biosensing. Shao et al. [5] compared the electroanalytical properties of NG electrode with pure graphene (G) and Pt/C electrode. Oxygen reduction reaction (ORR) overpotential is lower on NG than G, meaning that N doping significantly increases the electrocatalytic activity of graphene towards ORR. Although the NG electrode exhibited a lower initial electrocatalytic activity than Pt/C, it was much more stable and durable, suggesting that it may be possible to replace expensive Pt with low-cost NG. Moreover, unlike Pt, ORR on NG was not influenced by fuel molecules, making NG in direct liquid fuel cells very promising. The overpotential during electrocatalytic H_2_O_2_ reduction was significantly reduced, and a well-defined and enhanced H_2_O_2_ reduction peak was observed around −0.2 V, demonstrating better NG electrocatalytic activity compared to undoped graphene for H_2_O_2_ sensing. Ruiyi et al. [6] incorporated nitrogen in multiple graphene aerogel/gold nanostars (N-doped MGA/GNS) and reported that such a sensor was more sensitive than that of all reported DNA sensors to date. Saengsookwaow et al. [7] showed that, during cyclic voltammetry, a screen-printed carbon electrode (SPCE) functionalized by NG/Polyvinylpyrrolidone PVP/Gold nanoparticles (AuNPs) increased the anodic peak current by a factor of 10 compared to unmodified SCPE, due to a significant improvement in the interfacial charge transfer. This type of electrode showed higher electrochemical sensitivity and electrocatalytic activity toward hydrazine oxidation, leading to successful determination of hydrazine content in fruit and vegetable samples. Recently, Li et al. [8] also studied SPCE functionalized by NG sheets (NGS) obtained from graphene oxide and reported consistent sensitivity, selectivity and stability (with less overpotential required for oxidation) for nicotine detection, including when the molecule was in urine and tobacco samples.

However, most previous studies were performed with NG synthetized using rather complex chemical routes, with limited control of the nitrogen concentration incorporated in the graphene network. Thus, the investigation of electrochemical properties of NG films obtained by more simple routes is still of great interest for the next generations of electrodes. In particular, synthesis of NG film in one step from a solid carbon source directly onto silicon electrodes has been less explored than NG films obtained by other routes, including CVD processes and various reduction processes of GO by thermal annealing, plasma treatment, hydrothermal or solvothermal reactions in the presence of a nitrogen precursor. In a previous paper [9], we reported on the performance of a graphene electrode processed in one step by pulsed laser deposition directly coupled with in situ thermal annealing (PLD-TA). The electrochemical behavior of the NG film was studied in the presence of the redox probe, ferrocene methanol, which was shown to be the most suitable for quantifying electron transfer with graphene. Cyclic voltammetry revealed excellent electrochemical kinetic and quasi-reversibility performances. The attachment of ethynyl aryl groups on the surface of the electrode was robust, paving the way for the specific attachment of molecules bearing an azide function using the click reaction.

Moving on from there, in the present study we investigated the electroanalytic performance of a NG-functionalized electrode obtained using the one-step PLD-TA process previously successfully used for the electroanalytical investigation of pure graphene film. In a recent review [10], we mentioned that only three papers reported on the synthesis of NG films from an amorphous nitrogenated carbon film (a-C:N) obtained by PLD. Kumar et al. [11] reported in situ growth of n-type NG films (2 at % of nitrogen) by PLD performed at 973 K, with an increase in electrical conductivity with increased nitrogen partial pressure. Ren et al. [12] used the same approach and obtained NG films (1.7 to 3.2 at %) showing improved chemical enhancement for Raman analysis of absorbed Rhodamine 6G molecules, compared to pristine graphene. Recently, our group reported for the first time the synthesis of NG-doped few-layer graphene from a solid state nitrogen carbide (a-C:N film) synthetized by femtosecond pulse laser ablation [13]. We investigated the nanostructure and chemical composition of an NG film obtained by a vacuum thermal annealing at 780 °C of an a-C:N film previously deposited onto a SiO_2_ substrate and further covered by a Ni catalytic film (150 nm). Here we optimize the previous protocol to form the NG film directly by high temperature condensation of the laser-induced carbon plasma plume in the presence of nitrogen atmosphere, onto the Si electrode previously covered by a Ni catalytic film. The structure and composition of the NG film, compared to undoped ones, were investigated by Raman spectroscopy, X-ray photoelectron spectroscopy (XPS) and scanning electron microscopy (FEG-SEM). To show the interest of using NG film as an electrode for biological applications, its performance in the detection of hydrogen peroxide (H_2_O_2_) was compared to that of graphene film. H_2_O_2_ is the product of the enzymatic detection of glucose in diabetes diagnosis and its electrochemical detection is implemented in a commercial glucometer. Moreover, H_2_O_2_ is involved in different signal transduction pathways and cell fate decisions. The “redox signaling” mechanism includes the H_2_O_2_-mediated reversible oxidation of redox sensitive cysteine residues in enzymes and transcription factors, thereby altering their activities. In comparison to normal cells, cancer cells are characterized by an increased H_2_O_2_ production rate and an impaired redox balance, thereby affecting the microenvironment as well as the antitumoral immune response [14]. There is consequently a strong demand for hydrogen peroxide detection in the cell environment.

## 2. Materials and Methods

### 2.1. Graphene Film Synthesis and Characterization

**Figure 1:**
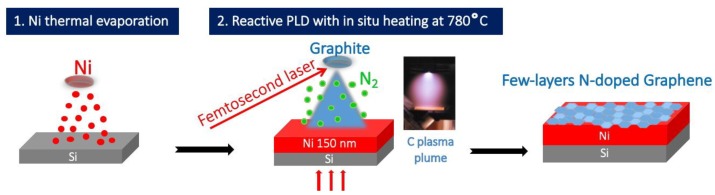
Synthesis of the N-doped graphene electrode.

Figure 1 is a schematic diagram of NG film synthesis. Nickel film (150 nm thick) was deposited by thermal evaporation on the top of an n-doped silicon substrate, previously cleaned (in acetone then in ethanol and DI water baths) in a vacuum chamber pumped at a base pressure of 10^−6^ mbar. High purity (99.99%) Ni was molten thermally in a tungsten nacelle and evaporated towards the substrate. The deposition rate was set at 1.5 nm/min to minimize residual stress in the growing film, thereby limiting film delamination. The Ni/Si samples were introduced in the PLD chamber pumped at a base pressure of 10^−6^ mbar, annealed at 780 °C for 30 min to increase the Ni grain size. While maintaining the temperature of 780 °C, carbon was ablated from a high purity graphite (99.9995%) target using a femtosecond laser (wavelength = 800 nm; pulse width = 60 ns, repetition rate = 1 kHz, energy density = 5 J/cm^2^) at a temperature of 780 °C. The Ni/Si substrates were mounted on a sample holder placed at a distance of 36 mm from the graphite target. Nitrogen gas (99.9995% purity) was introduced as a reactant gas in the vacuum chamber at a pressure of 10^−1^ mbar.

Undoped graphene films were also synthetized using the same protocol, but without introducing nitrogen gas during carbon ablation. The ablation time was adjusted to keep both carbon and nitrogenated carbon film thicknesses equal to 40 nm. In some cases, the temperature of 780 °C was maintained for a defined period after deposition to observe the effect of longer heating periods. The procedure ended with natural cooling of the samples, before opening the vacuum chamber. In the present paper, we selected four deposition conditions to highlight the major effects due to nitrogen doping, compared to undoped films. The conditions are summarized in Table 1.

The morphology of the sample was observed using a field emission gun scanning electron microscope (FEG-SEM) Novananosem 200, (FEI, Hillsboro, OR, USA) operated at 15 kV. Micro-Raman analyses were performed using an Aramis spectrometer (Horiba Jobin Yvon, Gières, France), with 442 nm (2.81 eV) excitation laser focused through a ×100 objective with high aperture, ensuring the micrometric resolution of the analysis, and allowing for precise Raman mapping of the samples.

For each sample, 20 × 20 µm^2^ areas were scanned, recording a Raman spectrum every 2 µm (giving a total of 100 spectra per scanned area). The laser power was kept below 3 mW to avoid damaging the surface of the film. Raman components were associated with a Lorentzian fit, safe for the asymmetric G peak, which was fitted using a Breit-Wigner-Fano function. A custom fitting function was computed for all recorded spectra, and a simple linear function was added to eliminate the background. This enabled access to the exact values of the various peak positions, widths and maximum peak height intensity. XPS analysis was performed at the SOLEIL Synchrotron (Saclay, France) on the ANTARES beam line. The ring operating conditions were 2.5 GeV electron energy, with injection currents of 500 mA and “Top-up” mode. Radiation was monochromatized using a plane-grating monochromator (PGM), which is characterized by a slitless entrance and the use of two varied linear spacing (VLS) gratings with variable groove depth (VGD) along the grating lines. The diameter of the X-ray spot impinging the surface is 140 μm and the X-ray energy was fixed at 700 eV for analysis of the graphene films. The photoemission spectra were taken with incident photon energies of 700 and 350 eV, with 190 meV and 140 meV energy resolution, respectively.

### 2.2. Electrochemical Measurements

Electrochemical measurements were carried out in a conventional one compartment three electrode cell with an internal volume of 5 mL. This electrochemical cell was designed to maintain a fixed distance between the electrodes. It was manufactured with two inlets, one for positioning the reference electrode and the other for injecting H_2_O_2_. This feature prevented further manipulation or movements of the electrodes (fixing the geometry of the cell and also ensuring the reproducibility of measurements). For this work, a saturated calomel electrode from Hach Lange (Marne-la-Vallée, France) was used as the reference electrode, a planar platinum electrode (0.59 cm^2^) was used as the counter electrode and the nitrogen doped and undoped graphene samples were the working electrodes. The active surface of the working electrode, determined by a polyethylene terephthalate (PTFE) O-ring seal, was 0.07 cm^2^. This three-electrode system was connected to a Bio-Logic potentiostat VMP2 (Bio-Logic Science Instruments, Seyssinet-Pariset, France). Results were recorded using EC-Lab software (v11.27) from Bio-Logic Science Instruments. In order to characterize the electron transfer rate for the different nitrogen doped and undoped graphene samples, cyclic voltammetry was performed in a 0.5 M 1,1′ ferrocene-dimethanol solution of 0.1 M NaClO_4_. The scan rate was 100 mV/s. The detection of H_2_O_2_ in a non-deaerated 0.1 M phosphate buffer saline (PBS) solution (pH 7.4) was detected through linear sweep voltammetry in the anodic range from 0 to 1000 mV with a scan rate of 100 mV/s.

## 3. Results

**Figure 2:**
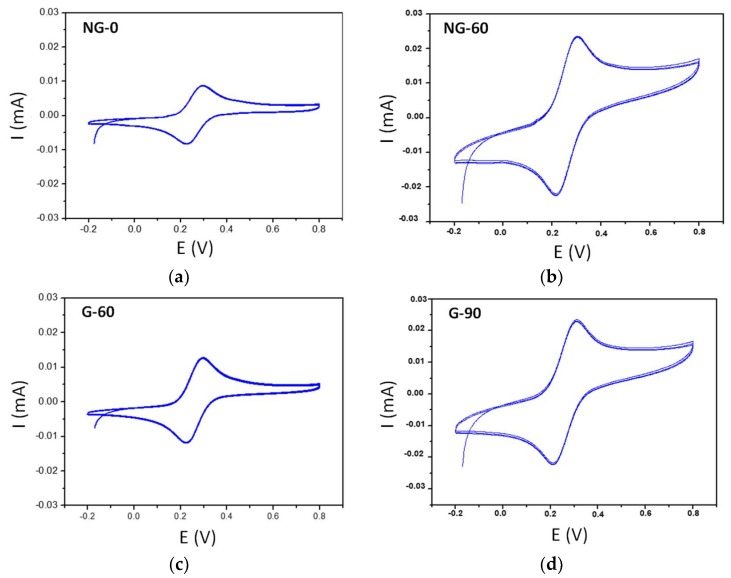
Cyclic voltammetry on (**a**) NG-0; (**b**) NG-60; (**c**) G-60 and (**d**) G-90 films, in a 0.5 M 1,1′ ferrocene-dimethanol solution of 0.1 M NaClO_4_. The scan rate was 100 mV/s.

Cyclic voltammetry measurements obtained with ferrocene methanol on pure graphene and NG films are presented in Figure 2, and the main results of the electrochemical measurements are listed in Table 2. The capacitive current appears to be higher with pure graphene film, due to the formation of more edge structures [15].

The length of the annealing time following graphene growth had no significant effect on the intensity of the anodic peak of ferrocene methanol. The value of ∆*E* between anodic and cathodic peaks of ferrocene methanol increased with an increase in annealing time for both graphene and NG films. For the NG-0 film, in the absence of annealing following growth, the value of ∆*E* was close to the theoretical value of 59 mV, showing the high reversibility of the redox probe.

**Figure 3:**
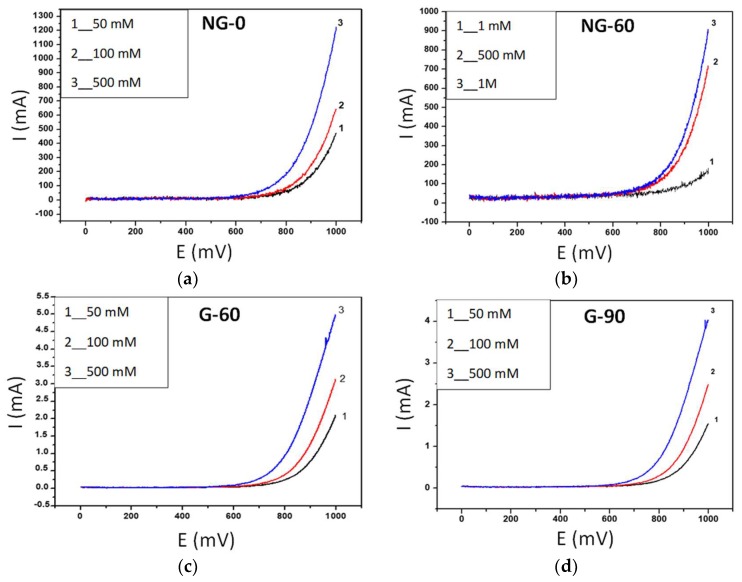
Linear sweep voltammetry of the (**a**) NG-0; (**b**) NG-60; (**c**) G-60 and (**d**) G-90 films, in the presence of different concentrations of H_2_O_2_ in 0.1 M PBS solution (pH 7.4).

The oxidation of hydrogen peroxide began at a potential value of 600 mV for both types of films, as shown in Figure 3. The values of the oxidation intensities, reported in Table 2 for 500 mM of hydrogen peroxide, were measured at a potential value of 1 V. Our results show that the NG-0 film has excellent electrocatalytic properties without additional annealing after graphene growth, leading to the high reversibility of the ferrocene methanol redox probe and high sensitivity for hydrogen peroxide detection, with a detection limit of 1 mM and an oxidation intensity 240 times higher for 500 mM of H_2_O_2_ than undoped G-60 film.

**Figure 4:**
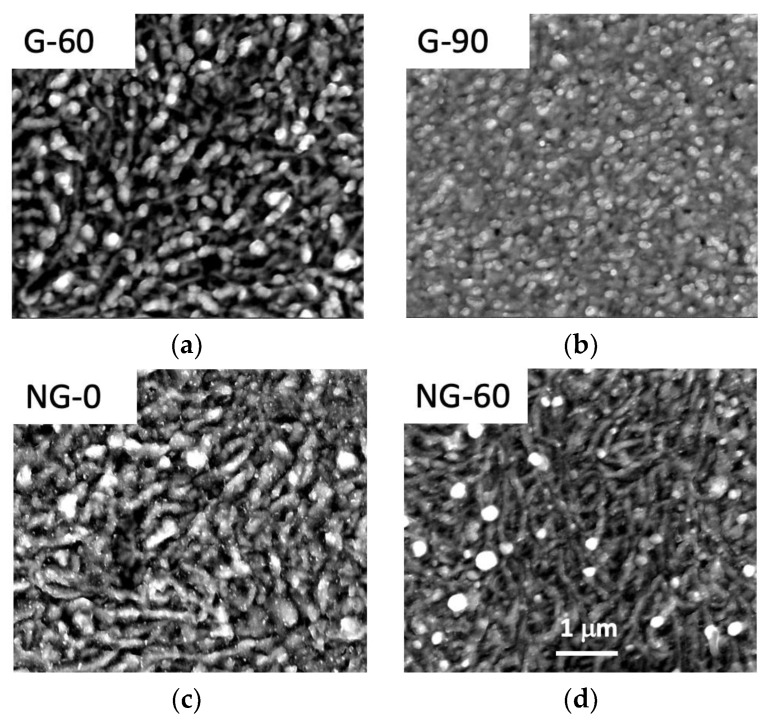
FEG-SEM images of (**a**) pure graphene with 60 mn post-deposition annealing; (**b**) pure graphene with 90 mn post-deposition annealing; (**c**) N-doped graphene with no post-deposition annealing; (**d**) N-doped graphene with 60 mn post-deposition annealing. The sub-micrometer texture was attributed to the texturing of the Ni catalyst film caused by thermal annealing. The four images are depicted with the same magnification 1 µm as noted on the image related to NG-60.

**Figure 5:**
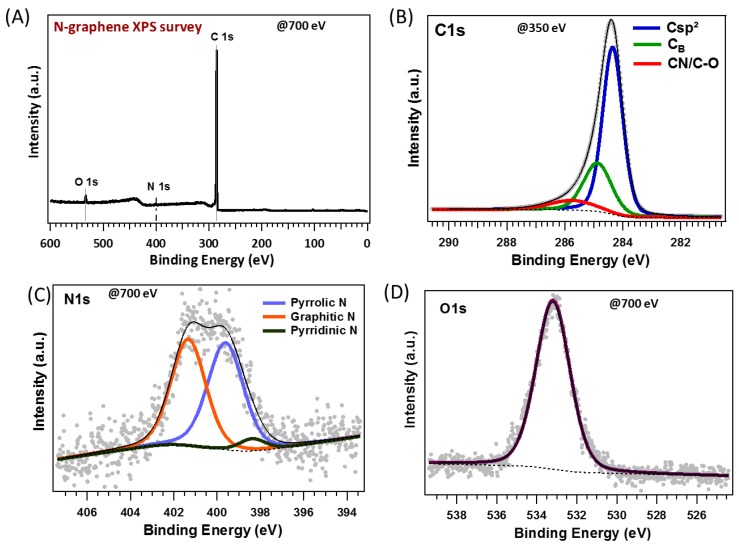
XPS spectra of the N-doped NG-0 graphene film; (**A**) XPS 700 eV overview spectrum; (**B**) XPS 350 eV C1*s* core level spectrum; (**C**) XPS 700 eV N1*s* core level spectrum; and (**D**) XPS 700 eV O1*s* core level spectrum.

The direct observation of the graphene-covered electrode by FEG-SEM (Figure 4) highlights a textured surface, with grain sizes in the range of 100 nm whatever the nature of the graphene film. Such a surface architecture is typical of graphene films synthetized on Ni films elaborated by thermal evaporation with subsequent Ni grain growth during the annealing process used to form the graphene films [9]. We do not observe any significant differences of morphology, with the FEG-SEM resolution at hand, between the four different graphene layers. Probably, the thermal heating during the PLD process is the crucial step inducing such a morphology, and the additional thermal annealing carried out on NG-60, G-60 and G-90 does not affect the surface morphology. Certainly, the nature and composition of the films, as deduced from XPS and Raman investigations, with such a significant difference between undoped and N-doped graphene films versus thermal annealing, is worth underlining. XPS was carried out on the NG-0 film obtained without post-annealing, given the significant electroanalytical result related to hydrogen peroxide oxidation. In Figure 5A, the XPS survey spectrum of the NG-0 film shows carbon located near 284 eV, nitrogen located near 400 eV, and some traces of oxygen near 533 eV.

The N/C intensity ratio deduced from the spectra was 0.01786, which is consistent with a nitrogen doping of 1.75 at %. Figure 5B shows deconvolution of the C1*s* into three components. The most intense component was centered at 284.4 eV and was attributed to *sp*^2^ hybridized C atoms in graphene. The two other ones were located at 284.9 and 285.8 eV, respectively. The component at 284.9 eV was attributed to disordered carbon (*C*_B_) and may be considered as an intermediate state between *sp*² and *sp*^3^ hybridizations that can be found in nano-diamond and amorphous carbon films, in agreement with References [16,17,18].

The component at 285.8 eV was attributed to C–N or C–O bonds [19,20]. However, it is known that the peak of C–O is overlaid with C–N; therefore, it may be difficult to discriminate between the C–N and C–O oxygen group [4,9,12].

**Figure 6:**
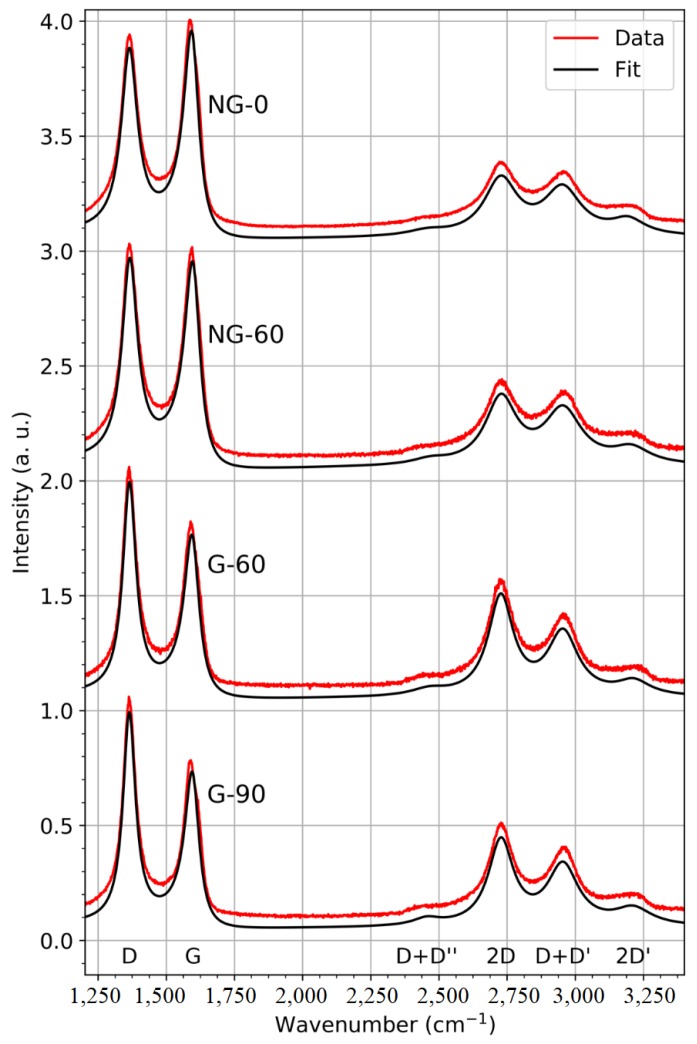
Typical Raman spectra of undoped and N-doped graphene films. The temperature during PLD graphene synthesis was 780 °C in all cases. Post-annealing times (min) are indicated in parentheses.

Raman results related to the undoped and doped graphene films are shown in Figure 6. All the samples had the bands traditionally found in Raman spectrometry graphene materials; these results are in agreement with those reported in References [21,22]. For graphene films, the D, G and 2D bands are the most significant for the characterization of the thin film structure. The G band is associated with covalent C–C bonding vibrations in the graphite matrix and is present in every carbon material containing *sp*^2^ bonding. The D band is associated with the pulsation of aromatic circles (“breathing mode”) and appears only in the presence of defects and dislocations in the graphitic matrix. The intensity ratio between the D band and G band (D/G) is thus an indication of disorder in the carbon structure. The 2D band is associated with a double resonance Raman scattering process between two aromatic circles. It appears in both graphene and graphite, and the intensity ratio of the 2D band versus the G band (2D/G) is a good indicator of the graphene-like quality of a thin film, a ratio higher than 1 being indicative of monolayer graphene. In non-defective graphene, the study of the 2D peak position and width is also a good way to count the number of layers and to identify their stacking configuration [22,23]. Additional D + D”, D + D’ and 2D’ bands are also observed in Figure 6. The D + D” and 2D’ peaks are, as the G and 2D peaks, usual features in most graphene samples [24]. They emerge, like the 2D peak, as a combination of two phonon mode individually associated with defects (D’ and D”) allowing so-called breathing of aromatic rings in carbon materials. The combination of those resonances can appear without defects as the two phonons can verify momentum conservation provided they have opposite wavevectors. In the case of the D + D’ band, also sometimes labelled as D + G, the comprehension of excitation mechanisms remains rather unclear, but whether it is a combination of D and G phonons or D and D’ does not change the fact that respectively one or both of the phonons need a defect to arise. Thus, the D + D’ band mostly appears in defective graphene-like material [25], which explains its presence in our materials.

**Figure 7:**
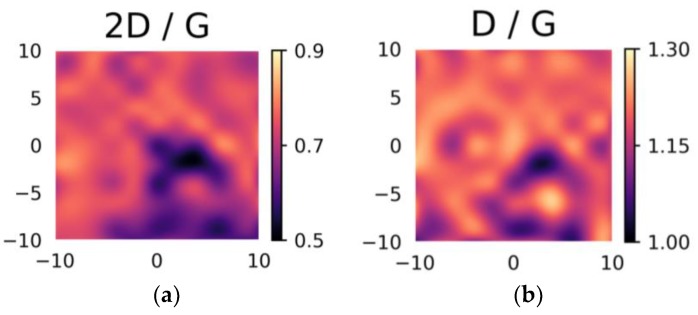
Maps of the Raman (**a**) 2D/G and (**b**) D/G intensity ratios recorded at the 442 nm excitation wavelength on a 20 × 20 µm^2^ area of the pure graphene film annealed at 780 °C for 90 min (G-90). *X*-*Y* scales in μm.

All the Raman spectra shown in Figure 6 present a higher D band compared to the G band, implying a very defective nature of the thin films. The 2D peak maximum was always lower than the G peak maximum, which may be associated with the multilayer nature of the graphene. A reduction in the 2D band in N-doped samples should also be noted; this is expected when nitrogen is introduced in the graphene matrix [13], although one would also expect an increase in the D/G ratio [26], which was not the case here. Typical maps of peak ratios, like the one in Figure 7 related to the multilayer pure graphene sample obtained with 90 mn post-annealing, enable evaluation of the uniformity of the synthesized thin films. In the case of pure graphene, a relative lack of uniformity appears in both the 2D/G and D/G ratios, with respective variations from 0.5 to 0.9 and from 1.0 to 1.3. The lack of homogeneity at the micrometric scale is consistent with the defective nature of the film. A slight correlation between areas with low D/G ratio and low 2D/G ratio can be observed, consistent with a slightly higher number of defects in areas where there are fewer graphene layers.

**Figure 8:**
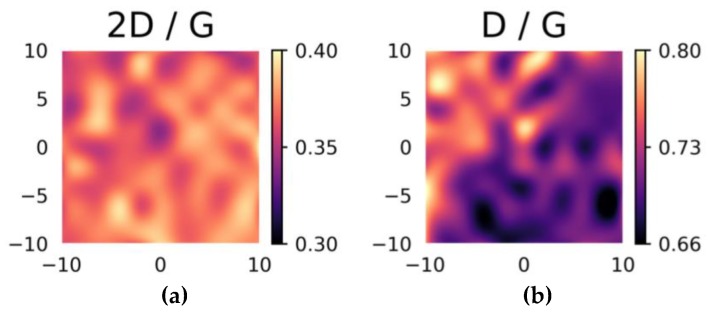
Maps of the Raman (**a**) 2D/G and (**b**) D/G intensity ratios recorded at an excitation wavelength of 442 nm on a 20 × 20 µm^2^ area of the N-doped graphene film with no post-deposition annealing (NG-0). *X*-*Y* scales in μm.

Figure 8 shows the impact of incorporating nitrogen in the sample synthesized at 780 °C with no post-annealing. The intensity ratio of the 2D and G peaks exhibit considerably less variation than in Figure 7, whereas the D/G intensity ratios still show high variability (note that in Figure 7 and Figure 8 the scales of the color bars are not the same) but are generally lower. This may mean that incorporating nitrogen helps to stabilize the multilayer graphene on the substrate, giving it a more organized structure.

The Raman findings are summarized in Table 3 where the average fit parameters obtained for scanned areas relate to the four samples. As mentioned above, we focused on the D, G and 2D peak parameters. The 2D/G and D/G intensity ratios further confirm the information provided by Figure 6. Pure graphene samples exhibited higher ratios of both the 2D/G and D/G bands than the N-doped graphene samples.

It should be noted that, in the course of this study, further post-annealing durations were tested in addition to those presented here. It was impossible to obtain any kind of pure graphene material without at least 30 min of post-deposition annealing. However, it was possible to synthesize an N-doped graphene film without post-annealing, i.e., the substrate was left to cool naturally immediately after the a-C:N pulsed-laser deposition. We would like to underline that this possibility implies an effect of the nitrogen environment on the catalysis of graphene growth by Ni, which opens the way for a more rapid fabrication of N-doped graphene. This also implies that, despite the high temperature used for graphene synthesis using annealing on a nickel catalyst, the as-deposited carbon structure may have a strong influence on the catalytic process. Additionally, it appears that lengthening the post-annealing period is an advantage with pure few-layer graphene, as the peak D/G ratio decreased and the 2D/G ratio increased when post-annealing time was increased from 60 to 90 min. This is in contrast to the fact that for N-doped graphene films, post-annealing only appeared to increase the D/G ratio, pointing to a higher number of defects.

All the observed peaks appear to be more intense than those of monocrystalline non-defective graphite or graphene. The full width at half maximum (FWHM) of the G peak is generally around 15 cm^−1^ [22] while here it was 70 cm^−1^. The width of this peak may be associated with the distortion of the C–C *sp*^2^ bonding angle. This is to be expected due to the nanotexturing of our samples, as observed by FEG-SEM shown in Figure 4. The position of the peak was also always upshifted here compared to graphite or monolayers graphene, i.e., between 1590 and 1595 cm^−1^ compared to 1582 cm^−1^ [22].

These characteristics, combined with the high D versus G peak intensities ratio, are clear indicators of highly nano-crystallized graphene-like layers. The broadening of all peaks due to the nanotexturing of the substrate during annealing makes it impossible to draw clear conclusions concerning the precise number of graphene layers by studying the 2D band Full Width Half Maximum (FWHM).

## 4. Discussion

The objective of this section is to discuss the significant improvement of electroanalytical oxidation of H_2_O_2_ by the N-doped graphene films compared to undoped ones in more detail. What is the main effect among the defective nature of the N-doped graphene films, the dopant concentration or the nature/proportion of the nitrogen chemical functions in the graphene network?

According to the previous section, the morphologies of all films (doped and undoped) appear to be textured, probably due to the texturing of the nickel catalyst surface caused by thermal annealing. We previously showed that the formation of nickel silicide contributed to texturing when graphene was grown on a silicon substrate covered by a Ni thin film [27].

Likewise, all films have a defective structure, as shown by Raman investigations, and nitrogen doping at a concentration as low as 1.6 at % does not significantly influence the defective structure of graphene, which is probably inherent to the synthesis process, as already observed by Schiros et al., who compared CVD synthesis of graphene and N-doped graphene films [28]. Moreover, when extended beyond the N-doped graphene synthesis, heat treatments do not cause significant changes to the nanoarchitecture of graphene film in terms of texture and number of graphene layers. Based on the comparison of the 2D/G and D/G ratio maps, the textured films are heterogeneous and the concentration of defects was slightly higher in areas where there are fewer layers of graphene.

However, a huge difference in electroanalytical oxidation of H_2_O_2_ was observed between undoped and N-doped graphene films, and the difference was more pronounced when the heat treatment was limited during the PLD film growth in the presence of nitrogen gas. The in situ nitrogen doping process during PLD graphene growth described in the previous section, led to the formation of a few-layer graphene film containing 1.75 at % of nitrogen, with a similar proportion of pyrrolic-N and graphitic-N, and a negligible amount of pyridinic-N. According to the literature, in particular, in Wang et al. [4] and in Shao et al. [5], the presence of incorporated nitrogen induces a change in the Fermi level, which is responsible for the doping effect and opens the band gap of the graphene structure, thus enhancing electrochemical reactivity. The high level of electronic state density and the efficient quantity of free electrons in N-doped graphene facilitates H_2_O_2_ oxidation. In particular, carbon atoms adjacent to nitrogen dopants may have a substantially positive charge density to counterbalance the higher electronic affinity of N atoms, consistent with the increased adsorption of H_2_O_2_ involved in the oxidation reaction. Such a mechanism has also been shown by density functional theory (DFT) simulating the physisorption process of H_2_O_2_ onto graphene-based surfaces [29]. Additionally, the structural defects resulting from N doping increased the amount of unsaturated carbon atoms located at graphene edge sites, which appeared to be very active in reacting with oxygen containing groups. During H_2_O_2_ electrocatalytic oxido-reduction, the O–O bond in H_2_O_2_ was more easily broken at the surface of N-doped graphene because N doping induced the charge delocalization of graphene. With our NG-0 film, in the absence of annealing following growth, the oxidation of H_2_O_2_ began at a potential value of 600 mV. This value is rather high compared to that reported by Wang et al. (200 mV), who observed a four times higher H_2_O_2_ oxidation signal with the NG films compared to the undoped ones [4]. Our results show that the NG-0 film presents excellent electrocatalytic properties leading to the high reversibility of the ferrocene methanol redox probe and high sensitivity for hydrogen peroxide detection, with a detection limit of 1 mM. A 240 times higher H_2_O_2_ oxidation signal was observed for the NG-0 film compared to the average value obtained with the undoped graphene films.

Such high electroanalytical reactivity is generally attributed both to chemical functionalization (both pyrrolic, pyridinic and graphitic nitrogen-carbon forms are generally reported in the literature) and to structural defects caused by nitrogen atoms. However, based on our experiments, the nature and proportions of the various N–C chemical functions certainly play a more significant role in the electroanalytical oxidation of H_2_O_2_ than the number of defects, which is similar with or without N doping. Such an influence was already highlighted by Xu et al. [3]. According to these authors, the relationship between the nature of N functions and the properties of N-doped graphene needs to be clarified, so that more desirable properties for specific applications can be selected. Based on experimental and theoretical (DFT calculations) considerations [28,30], the nature and level of doping in N-doped graphene depends on the proportion of the three main functional groups: graphitic-N is responsible for an n-doping effect and pyridinic-N and pyrrolic-N are responsible for a p-doping effect. The balance between the three chemical functions may strongly affect the electroanalytical performance of the N-doped graphene. More precisely, nitrogen in the graphitic-N configuration has 4 of the 5 electrons filling in the σ- and π-orbitals, leaving one extra electron. About 50% of the additional charge is localized on the N dopant coupled with its nearest carbon neighbors, whereas the remaining 50% is distributed in the local network of carbon π-states, thus inducing n-doping and preserving high electron mobility. Pyridinic-N and pyrrolic-N dopants have the opposite electronic effect, as they withdraw charge from their carbon neighbors. In the case of pyridinic-N, two electrons fill σ bonds with carbon neighbors, and two electrons form a lone pair in the graphene plane. The remaining electron occupies the nitrogen π-state. As a consequence, pyridinic-N is the equivalent of a nominal carbon in graphene, but an π electron is missing due to the vacant site, hence p-doping the graphene. Our NG-0 film contained 49% of graphitic-N and 47% of pyrrolic-N, whereas pyridinic-N contents were as low as 4%. With a 1.35 at % N content close to our doping concentration (1.75 at %), Wang et al. [4] obtained a nitrogen-doped graphene film with significantly higher proportions of pyrrolic-N and pyridinic-N and a lower proportion of graphitic-N than we obtained. We conclude that our nitrogen doping process induced a rather higher proportion of mixed graphitic-N and pyrrolic-N than other N-doped graphene films reported in the literature, and this may be responsible for the very high electroanalytical H_2_O_2_ oxidation performance. However, our results do not follow the reactivity scale simulated by DFT by Wu et al. [29] who concluded that H_2_O_2_ oxidation reactivity occurred in the following order: pyridinic-N > Pyrrolic-N > Graphitic-N > Pristine graphene. However, it is difficult to compare DFT calculations performed with only one nitrogen-based function, even if true, with an experimental system comprised of a mixture of nitrogen-based functions embedded in a textured few-layer graphene material. Further studies are thus recommended to optimize N-doped graphene films with better control of the N-based chemical functions, in particular with distinct proportions of the various N-based functions, in order to confirm their effect as key factors for electrochemical applications of N-doped graphene films.

## 5. Conclusion

Here we report on experimental work on the electroanalytical performance of N-doped graphene silicon-based electrodes, obtained in one step by pulse laser deposition of carbon performed in a vacuum at high temperature, in the presence of a surrounding nitrogen atmosphere. The main conclusions are the following:

- The electrode is covered by a few-layer defective and textured N-doped graphene film, containing 1.75 at % of nitrogen distributed in both graphitic-N and pyrrolic-N chemical functions at similar proportions.
- With the ferrocene methanol system, the electrode displays excellent reversibility, 60 mV, close to the theoretical value of 59 mV, and very high sensitivity for hydrogen peroxide oxidation characterized by an intensity 240 times higher than that obtained with undoped graphene synthetized using the same process, and a detection limit of 1 mM of hydrogen peroxide.
- These significant electroanalytical results are correlated with the amount of N doping and with the proportion of both graphitic-N and pyrrolic-N chemical functions incorporated into the defective and textured few-layer graphene film.
- Additional heat treatment following the deposition process does not significantly modify the nanoarchitecture of the N-doped graphene films and slightly decreases the electroanalytical performance in terms of reversibility and hydrogen peroxide oxidation performance, compared to the N-doped films obtained with no additional heating.

Further works are recommended to achieve better control of the different N-based chemical functions embedded in the graphene network and to quantify their effects on the electroanalytical performances of N-doped graphene electrodes.

## Figures and Tables

**Table 1:** Specific deposition parameters of the graphene films.

Graphene Films	N_2_ Pressure during Deposition at 780 °C	Additional Period of Annealing at 780 °C after Deposition
NG-0	10^−1^ mbar	No additional annealing
NG-60	10^−1^ mbar	60 min
G-60	–	60 min
G-90	–	90 min

**Table 2:** Results of electrochemical measurements on NG and pure graphene films.

Graphene Films	Anodic Peak Intensity	∆*E* between Anodic and Cathodic Peaks	Intensity for 500 mM H_2_O_2_
NG-0	4.0 µA	60 mV	1200 µA
NG-60	10 µA	78 mV	700 µA
G-60	5.7 µA	65 mV	5 µA
G-90	8.7 µA	82 mV	4 µA

**Table 3:** Average Raman fit parameters and parameter ratios for four undoped and doped multilayer graphene samples with different post-annealing durations. The standard deviation for each parameter is in parentheses.

Samples	Intensity Ratio (Standard Deviation)		Peak Position (cm^−1^) (Standard Deviation (cm^−1^))			Peak Full Width Half Maximum (cm^−1^) (Standard Deviation (cm^−1^))		
	2D/G	D/G	D	G	2D	D	G	2D
NG-0	0.369 (0.011)	0.722 (0.032)	1365.6 (0.7)	1589.8 (1.7)	2721.7 (1.9)	78.2 (2.0)	73.9 (0.8)	139 (3.2)
NG-60	0.386 (0.011)	0.903 (0.026)	1366.8 (0.6)	1595.0 (1.00)	2724.4 (1.9)	69.5 (2.1)	71.0 (1.4)	129.0 (5.0)
G-60	0.659 (0.036)	1.242 (0.029)	1365.6 (0.8)	1594.5 (1.0)	2727.0 (1.7)	59.5 (1.4)	68.1 (2.1)	111.1 (2.6)
G-90	0.712 (0.060)	1.183 (0.042)	1365.9 (0.8)	1591.9 (1.7)	2726.2 (2.0)	60.4 (5.2)	66.8 (3.1)	108.4 (4.6)
